# Control-oriented unified modeling and hierarchical control of a cascaded DAB–Čuk converter for bidirectional battery charging

**DOI:** 10.1038/s41598-026-51180-z

**Published:** 2026-05-04

**Authors:** Rajat Kumar Samantaray, Laxmidhar Senapati, Pradyumna Behera, Ashwin Kumar Sahoo, Zefree Lazarus Mayaluri, Aswini Kumar Samantaray

**Affiliations:** 1 Department of Electrical Engineering, C. V. Raman Global University, Bhubaneswar, India; 2 Department of Electrical Engineering, Biju Patnaik University of Technology, Rourkela, India; 3 Department of Electronics and Communication Engineering, Manipal Institute of Technology Bengaluru, Manipal Academy of Higher Education, Manipal, India

**Keywords:** Cascaded converters, Dual Active Bridge (DAB), Čuk converter, single-phase-shift modulation, small-signal modeling, bidirectional power flow, Energy science and technology, Engineering

## Abstract

This paper presents a control-oriented modeling and validation study of a cascaded Dual Active Bridge (DAB)–Čuk converter for bidirectional battery-charging applications. The proposed topology combines the galvanic isolation and bidirectional power-transfer capability of the DAB stage with the continuous-current behavior and output-conditioning capability of the Čuk stage. Because the two stages are coupled through a finite intermediate DC-link capacitor, variations in DAB phase shift and Čuk duty ratio jointly influence the converter dynamics and complicate closed-loop design. To address this issue, a unified large-signal averaged model is first developed and then linearized to obtain a six-state small-signal representation of the cascaded system. The resulting model captures the dynamic interaction between the isolated front-end and the downstream DC–DC stage and provides a control-oriented basis for hierarchical regulation of the DC-link and battery-side variables. On this basis, a dual-loop control structure is implemented, in which the inner loop regulates the intermediate DC-link voltage through single-phase-shift (SPS) modulation and the outer loop regulates the battery-side current through duty-ratio control of the Čuk stage. The modeling and control framework is evaluated using MATLAB/Simulink studies and laboratory-scale experimental tests under reported bidirectional operating conditions. At the nominal G2V operating point, the analytical/simulation/experimental values of the intermediate DC-link voltage were 30.0/30.0/29.5 V, the battery-side voltage was 24.0/24.1/23.8 V, and the leakage-current peak was 9.0/9.2/8.5 A. These results provide local experimental support for the proposed unified modeling framework as a control-oriented description of the cascaded converter under the tested laboratory conditions, while broader transient validation, wider operating-range assessment, and parasitic-aware modeling remain subjects for future work.

## Introduction

Efficient bidirectional DC–DC conversion is a key requirement in electric-vehicle charging, energy-storage interfaces, renewable-energy systems, and DC microgrids. Among isolated converter topologies, the Dual Active Bridge (DAB) has attracted sustained attention because it combines galvanic isolation, high power density, and bidirectional power-transfer capability within a compact high-frequency structure^1,2^. Owing to its phase-shift-based operation, the DAB is particularly attractive in applications that require controllable power reversal without major changes in switching frequency or hardware configuration^3–6^. Recent studies have therefore considered DAB-based interfaces for hybrid renewable systems, battery-coupled energy-storage configurations, and EV charging applications^7–12^.

In many practical charging and storage interfaces, however, the isolated front-end converter alone is not sufficient to achieve the required downstream voltage conditioning or battery-side current regulation. Cascaded and multiport architectures therefore remain of continued interest, particularly when an isolated power-transfer stage is followed by an auxiliary DC–DC stage that performs downstream conditioning and battery-side regulation^13–17^. In this context, the cascaded DAB–Čuk configuration is of interest because it combines the bidirectional isolated power-transfer capability of the DAB with the continuous-current characteristics and output-conditioning flexibility of the Čuk stage. Such a structure is potentially useful in laboratory-scale bidirectional battery interfaces where both isolation and regulated battery-side operation are required.

A central challenge in cascaded converter systems is that the constituent stages do not operate independently. In particular, the DAB phase-shift command and the downstream duty-ratio command interact through the intermediate DC-link, so that variations in one stage alter the operating condition seen by the other. While prior studies have reported DAB steady-state analysis, SPS and extended phase-shift control, and closed-loop battery-charging implementations^2–4,6,9^, the coupled dynamics of an isolated DAB front end and a downstream regulation stage are often simplified in control-oriented analysis. Likewise, state-space averaging has long been used for controller-oriented modeling of PWM converters^18^. However, prior work has generally emphasized standalone DAB analysis, modulation strategies, or application-specific implementations, whereas low-order unified control-oriented modeling that explicitly retains DC-link coupling for hierarchical regulation of a cascaded DAB with downstream battery-side conditioning remains less developed.

To position the contribution more explicitly against the closest strands of prior work, Table 1 summarizes the main gap addressed here. The comparison is intentionally restricted to the most relevant studies already cited in this manuscript, namely standalone DAB steady-state and modulation studies^2,6^, charging-oriented DAB implementations^9–12^, broader cascaded and multiport interface studies^13–17^, and the generic state-space-averaging methodology used for PWM converters^18^. Across these strands, the literature largely emphasizes either standalone DAB behavior, application execution, or broader multiport structures, whereas an explicitly coupled low-order unified model for a cascaded isolated DAB–Čuk interface with hierarchical DC-link and battery-current regulation remains less developed.

**Table 1:** Closest-prior-work positioning of the present manuscript.

References	Topology/strand	Structure	Model/control emphasis	Explicit DC-link coupling	Main limitation relative to this work
^2,6^	Standalone DAB steady-state and SPS/DPS studies	Standalone	Steady-state/modulation; phase-shift control	No	No downstream regulation stage and no unified cascaded small-signal model.
^9–12^	DAB charging and EV-charger implementations	Mostly standalone charger interfaces	Application-oriented design/implementation; charging-oriented SPS/modulation	No	Focused on charger execution/design rather than a coupled DAB–Čuk control-oriented model.
^13–17^	Cascaded/multiport regulated interfaces	Cascaded or multiport	Topology and application studies; control varies by application	Usually not for this specific problem	Not targeted to an isolated cascaded DAB–Čuk battery interface with hierarchical DC-link/current regulation.
^18^	PWM state-space averaging methodology	Generic	Averaged small-signal framework; generic duty-ratio control	Not DAB-specific	Provides the modeling tool, but not the isolated bidirectional DAB coupling problem addressed here.
This work	Cascaded DAB–Čuk bidirectional battery interface	Cascaded	Unified averaged + six-state small-signal model; hierarchical DC-link and battery-current regulation	Yes	Local operating-point validation only; broader operating-range characterization remains future work.

The entries summarize the dominant emphasis of the cited literature strands used in this paper; the table is intended to clarify the specific modeling and control gap addressed here rather than to claim exhaustive literature coverage.

In light of Table 1, the contribution of the present manuscript is threefold. First, it develops a unified large-signal averaged model and a corresponding six-state small-signal model that retain the intermediate DC-link coupling between the isolated DAB stage and the downstream Čuk stage. Second, it uses that coupled model to interpret a hierarchical dual-loop controller in which the DAB regulates the intermediate DC link through SPS modulation and the downstream Čuk stage regulates the battery-side current through duty-ratio control. Third, it supports the proposed control-oriented description through nonlinear simulation and laboratory-scale bidirectional experiments, while explicitly restricting the claims to local operating-point validation under the reported laboratory conditions rather than full operating-range charger qualification.

The manuscript therefore evaluates local model consistency, bidirectional operation, and controller feasibility under the reported laboratory conditions rather than full-range charger characterization. The remainder of the paper is organized as follows. Section 2 describes the cascaded converter structure and operating assumptions. Section 3 summarizes the SPS operating principle of the DAB stage. Section 4 develops the averaged and small-signal models and presents the hierarchical control framework. Section 5 reports simulation results, Section 6 presents laboratory-scale experimental validation, and Section 7 concludes the paper.

## Proposed converter structure

Principal symbols used throughout the manuscript are summarized in Table 2.

**Table 2:** Principal symbols used in the manuscript.

Symbol	Description
$V_g$	Input DC source voltage
$V_s$	Intermediate DC-link voltage
$V_o$	Battery-side terminal voltage
$I_o$	Battery-side current; positive during G2V charging and negative during V2G operation
$V_P, V_S$	Instantaneous bridge-voltage levels applied by the primary and secondary DAB bridges
$v_p, v_s$	Instantaneous primary-side and secondary-side voltages in the interval analysis
$v_{p,\textrm{avg}}$	Averaged DAB bridge voltage used in the large-signal model
$L_k$	DAB leakage inductance
$C_s$	Intermediate DC-link capacitance
$L_1, L_2$	Čuk-stage input and output inductances
$i_{L_2}$	Čuk output-inductor current; used as the model representation of the battery-side current under CCM
$I_i$	DAB input current measured at the front-end converter input during the experimental steady-state test
$C_1, C_2$	Čuk-stage energy-transfer and output capacitances
$R_o$	Equivalent battery/load resistance used in the averaged and small-signal model
$\eta$	Lumped efficiency factor appearing in the DAB power-transfer relation
$\omega$	Switching angular frequency of the DAB stage
$T_S$	Switching period used in the interval and averaging analysis
${\mathbf{f}}_{\textrm{on}},{\mathbf{f}}_{\textrm{off}}$	ON-state and OFF-state vector fields of the downstream averaged model
$\phi$	DAB phase-shift angle
$D_{S1}$	Čuk duty ratio

The abbreviations CCM, G2V, V2G, PM, BW, and LPF denote continuous conduction mode, grid-to-vehicle operation, vehicle-to-grid operation, phase margin, bandwidth, and low-pass filter, respectively.

**Fig. 1:**
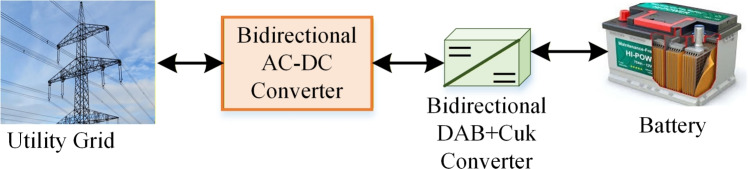
System-level block diagram of the proposed cascaded DAB–Čuk converter showing the isolated DAB front end, intermediate DC-link stage, downstream Čuk converter, and hierarchical control loops in which the inner loop regulates the DC-link voltage $V_s$ and the outer loop regulates the battery-side current $I_o$.

**Fig. 2:**
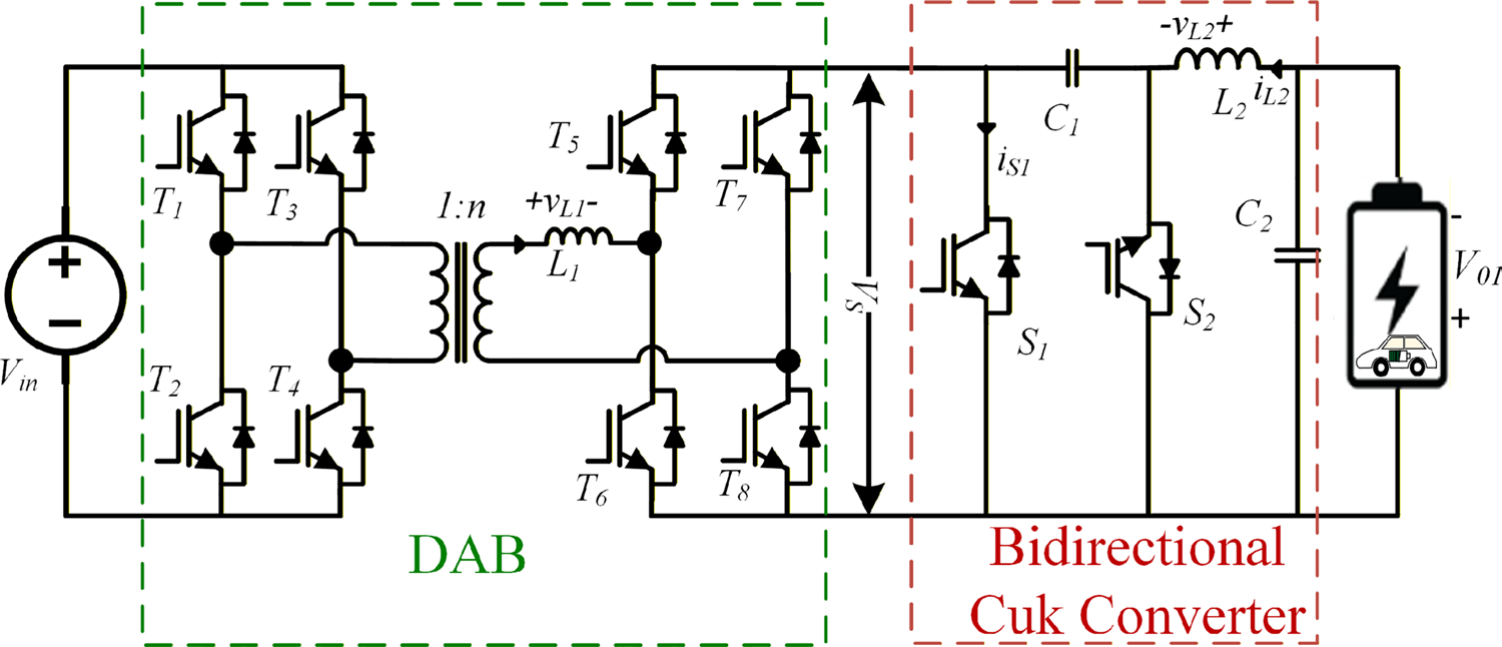
Circuit topology of the proposed bidirectional cascaded DAB–Čuk converter, including the isolated DAB stage, intermediate DC-link capacitor, and downstream Čuk stage used for battery-side current control and battery-terminal voltage conditioning.

The proposed converter combines an isolated Dual Active Bridge (DAB) front end with a downstream inverting Čuk converter, as shown in Fig. 1 and Fig. 2. The DAB stage provides galvanic isolation and controllable bidirectional power transfer between the input source and an intermediate DC-link capacitor, whereas the downstream Čuk stage performs battery-side conditioning and regulation. This cascaded arrangement is motivated by the broader use of isolated bidirectional converters in storage and charging interfaces^1,2,7,9–11^ and by continuing interest in multiport and cascaded DC–DC structures for regulated renewable-energy and battery-connected systems^13–17^. In the present work, the two stages are modeled as a unified system because the intermediate DC-link capacitor dynamically couples the DAB power-transfer process to the downstream regulation stage. In the sequel, $L_k$ denotes the DAB leakage inductance, whereas $L_1$ and $L_2$ denote the downstream Čuk-stage inductive elements.

### Dual Active Bridge stage

The DAB converter consists of two full bridges coupled through a high-frequency transformer of turns ratio $n{:}1$. The primary-side bridge is connected to the input DC source $V_g$, whereas the secondary-side bridge feeds the intermediate DC-link capacitor $V_s$, which serves as the input to the downstream Čuk stage. Power transfer through the transformer leakage inductance $L_k$ is regulated by the phase-shift angle $\phi$ between the primary and secondary bridge voltages. Under the ideal assumptions adopted for the analytical development and neglecting parasitic losses, the average transferred power can be expressed as

1 $$\begin{aligned} P_{\textrm{DAB}}=\frac{\eta V_g V_s}{\omega L_k}\,\phi \left( 1-\frac{\phi }{\pi }\right) \end{aligned}$$

where $V_s$ is the DC-link voltage on the secondary side and $\omega$ is the switching angular frequency. The DAB stage is used here as the isolated energy-transfer interface. Its role in the cascaded system is to regulate the intermediate power exchange while maintaining galvanic isolation and enabling reversal of power flow through the sign of the phase shift.

### Čuk stage

The downstream Čuk converter is connected to the DC-link output of the DAB stage and provides the battery-side conditioning function. The converter employs input inductor $L_1$, energy-transfer capacitor $C_1$, output inductor $L_2$, and output capacitor $C_2$ to regulate the battery-side current and establish the corresponding battery-terminal voltage across the equivalent battery/load representation $R_o$. Owing to its continuous input and output currents, the Čuk stage is suitable for cascaded applications where large current ripple at the DC link or at the battery terminals is undesirable. In the present topology, the Čuk stage is operated through pulse-width modulation with duty ratio $D_{S1}$, and under the sign convention adopted in this manuscript its steady-state voltage-conversion magnitude is given by

2 $$\begin{aligned} |V_o|=\frac{D_{S1}}{1-D_{S1}}\,V_s \end{aligned}$$

The Čuk stage is inverting with respect to the source-side reference; accordingly, Eq. (2) is written in terms of the steady-state voltage-conversion magnitude, while the direction of power flow is represented separately through the sign convention adopted for $I_o$ and $i_{L_2}$. Under CCM steady state, setting the derivatives of the averaged equations in Section 4 to zero gives $v_{C_1}=v_s/(1-D_{S1})$ and $|v_o|=D_{S1}|v_{C_1}|$, which lead directly to Eq. (2). A switched-model derivation of the downstream stage is summarized in Appendix A.

The Čuk topology is selected here because its continuous input and output currents help reduce ripple and improve output filtering, which is beneficial when the converter is supplied from a finite intermediate DC link.

### Cascaded operation and hierarchical control

In the proposed arrangement, the DAB stage governs the power transferred to or from the intermediate DC link, while the downstream Čuk stage regulates the battery-side current under the operating conditions considered in this work. Both stages are designed to operate in continuous conduction mode (CCM), which simplifies the averaged modeling and is consistent with the low-ripple operating objective of the cascaded topology.

In the present implementation, the regulated battery-side variable is battery current $I_o$. Positive $I_o$ denotes source-to-battery charging current during G2V operation, whereas negative $I_o$ denotes battery-to-source current during V2G operation. Similarly, positive DAB phase shift corresponds to net power transfer from the source side toward the battery side, while negative phase shift corresponds to reverse power transfer.

For the purposes of the averaged and small-signal model developed later, the battery-side current is represented by the Čuk output-inductor current $i_{L_2}$. Under the CCM operating condition considered here, $i_{L_2}$ provides the model variable corresponding to the controlled battery current $I_o$, whereas $V_o$ denotes the battery-terminal voltage reported in the steady-state results.

The DAB stage is controlled through single-phase-shift (SPS) modulation, in which the phase displacement between the primary and secondary bridge voltages determines the magnitude and direction of transferred power. Positive phase shift corresponds to power transfer from the source side toward the battery side, whereas reversal of the phase shift reverses the net direction of power flow. Thus, during grid-to-vehicle (G2V) operation, the DAB transfers power from the DC source to the battery-side stage, while during vehicle-to-grid (V2G) operation the transferred power direction is reversed.

The downstream Čuk stage is controlled through duty-ratio variation in order to regulate the battery-side current under charging and discharging conditions. Because the two stages interact through the finite DC-link capacitor, the overall control system is treated as a hierarchical structure rather than as two fully independent regulators. In the implementation considered here, the inner loop acts on the DAB phase shift to regulate the intermediate DC-link voltage $V_s$, while the outer loop adjusts the Čuk duty ratio to regulate the battery-side current $I_o$ represented in the model by $i_{L_2}$. This bandwidth-separated arrangement provides a practical framework for hierarchical coordination of the two control actions and establishes the control interpretation used in the unified small-signal model developed in Section 4.

The charging results reported in this work are confined to the constant-current interval, since the present emphasis is on control-oriented modeling and validation of battery-current regulation in the cascaded converter. A full CC–CV charging implementation would further require supervisory transition logic, voltage-limit enforcement, and battery-state-dependent mode switching. These charger-level functions are important in practical deployment, but they are beyond the local modeling and regulation scope considered here.

## Operating principle under SPS modulation

**Fig. 3:**
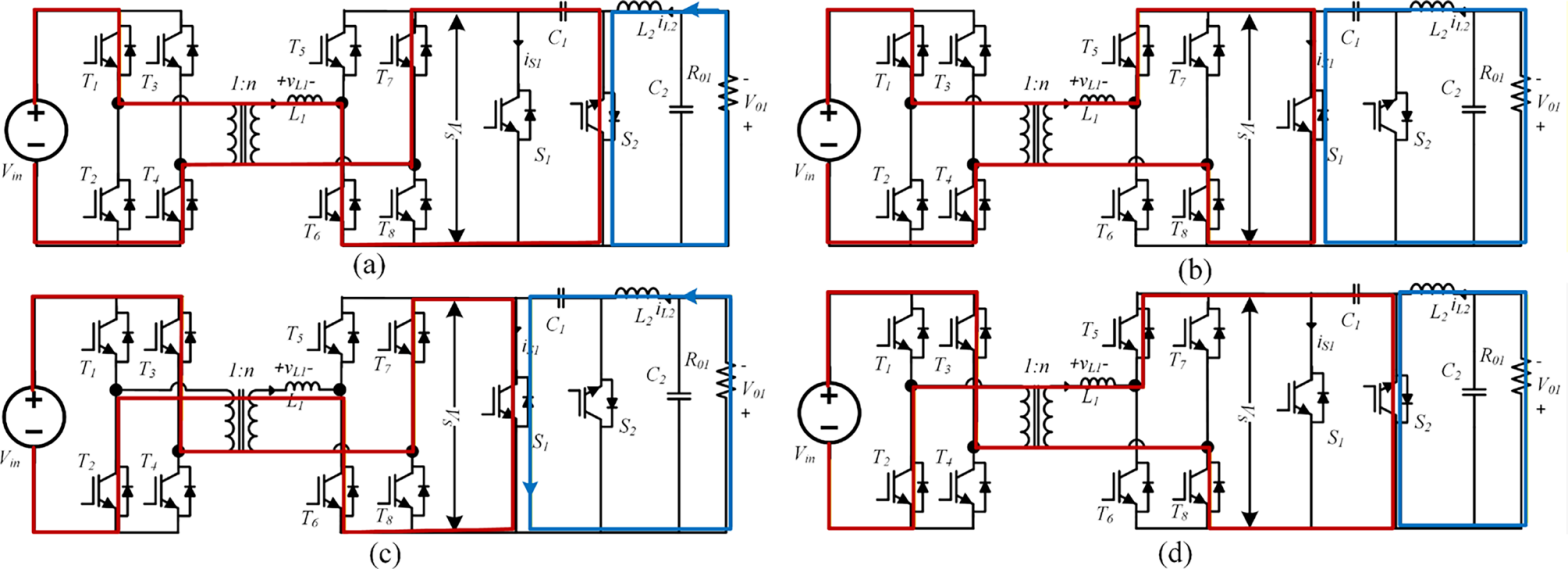
Switching sub-intervals of the cascaded DAB–Čuk converter under SPS modulation: (**a**) Sub-interval-1, (**b**) Sub-interval-2, (**c**) Sub-interval-3, and (**d**) Sub-interval-4.

**Fig. 4:**
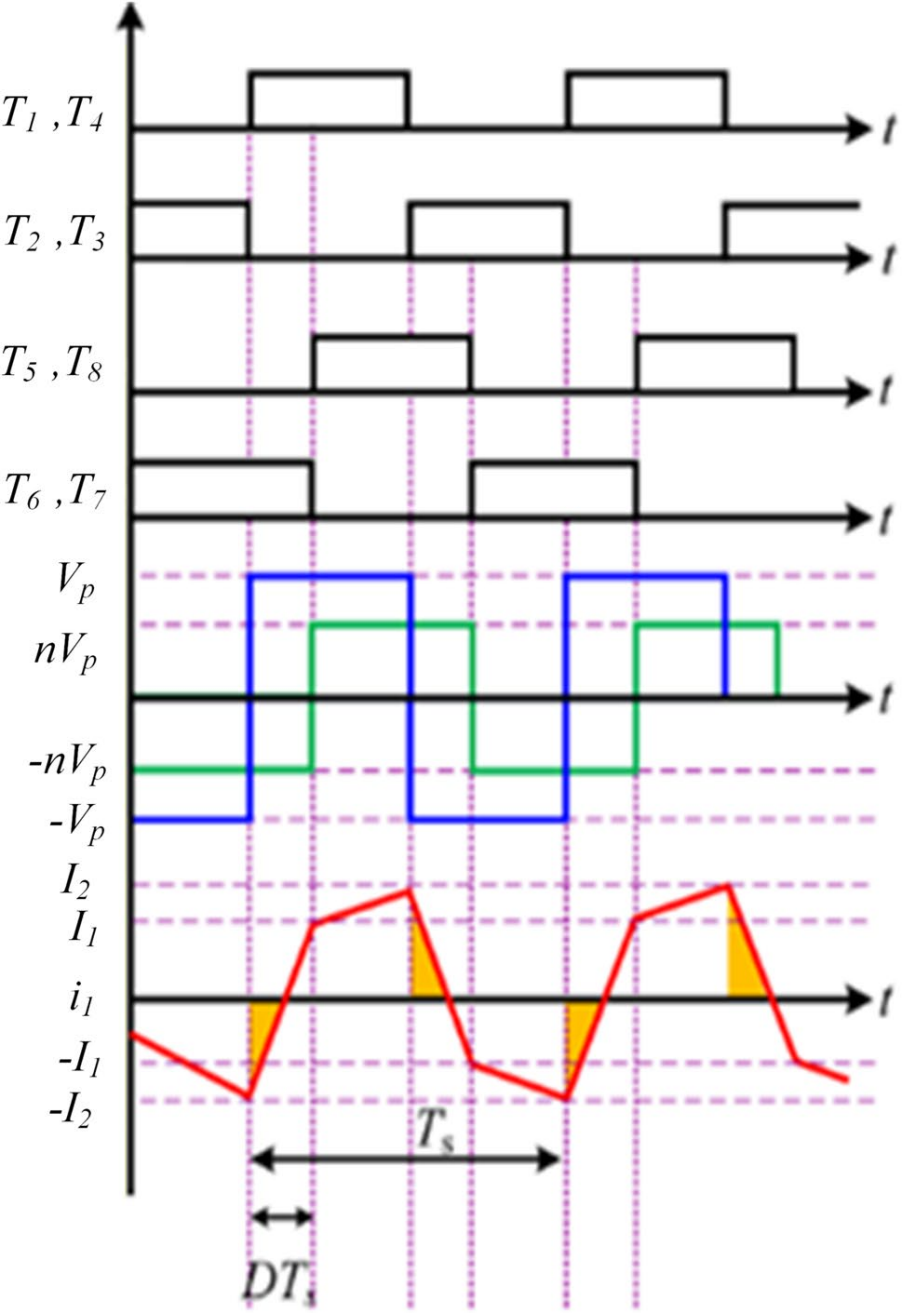
Idealized SPS waveforms of the DAB stage showing primary and secondary bridge voltages and the corresponding leakage-inductor current over one switching cycle.

The DAB stage operates under single-phase-shift (SPS) modulation, in which the phase displacement between the primary and secondary bridge voltages determines the average power transferred through the transformer leakage inductance. SPS operation is widely used because it provides a comparatively simple bidirectional control mechanism and has been studied extensively for steady-state analysis, closed-loop implementation, and charging-oriented DAB operation^2–4,6,9^. Over one switching period, the bridge states produce four operating sub-intervals, each associated with a distinct voltage difference across the transformer leakage inductance $L_k$ and hence a distinct slope of the leakage-inductor current. Figures 3 and 4 summarize these switching states and the corresponding idealized voltage and current waveforms.

In this section, uppercase symbols $V_P$ and $V_S$ denote the instantaneous bridge-voltage levels applied by the primary and secondary bridges, respectively, whereas lowercase symbols denote instantaneous variables. Bar and hat notation are reserved for the averaged and perturbed quantities introduced in Section 4 The interval-wise analysis presented below establishes the physical basis of the averaged DAB input representation used later for control-oriented modeling.

### Sub-interval-1 $(0 \le t < t_1)$

During the first interval, switches $T_1$ and $T_4$ conduct on the primary side, while $T_6$ and $T_7$ conduct on the secondary side, as shown in Fig. 3a. The applied bridge voltages are positive on both sides, producing a constant voltage difference across the leakage inductance. The instantaneous voltages can be written as

$$\begin{aligned} v_p=+V_P,\qquad v_s=+V_S,\qquad v_p'=\frac{v_p}{n}, \end{aligned}$$

where $n$ is the transformer turns ratio and $v_p'$ is the primary voltage referred to the secondary side. The leakage-inductor current $i_{L_k}$ therefore varies linearly according to

3 $$\begin{aligned} \frac{di_{L_k}}{dt}=\frac{V_P/n - V_S}{L_k} \end{aligned}$$

### Sub-interval-2 $(t_1 \le t < t_2)$

In the second interval, the primary bridge remains in the same conduction state, while the secondary bridge reverses polarity, with $T_5$ and $T_8$ conducting, as shown in Fig. 3b. The instantaneous voltages become

$$\begin{aligned} v_p=+V_P,\qquad v_s=-V_S,\qquad v_p'=\frac{v_p}{n}, \end{aligned}$$

and the current slope becomes

4 $$\begin{aligned} \frac{di_{L_k}}{dt}=\frac{V_P/n + V_S}{L_k} \end{aligned}$$

This interval corresponds to the main power-transfer phase, in which energy is transferred through the leakage inductance from the primary side toward the secondary side.

### Sub-interval-3 $(t_2 \le t < t_3)$

In this interval, both the primary and secondary bridges invert polarity. Switch pairs $T_2$ and $T_3$ conduct on the primary side, while $T_5$ and $T_8$ continue on the secondary side, as shown in Fig. 3c. The corresponding voltages are

$$\begin{aligned} v_p=-V_P,\qquad v_s=-V_S,\qquad v_p'=\frac{v_p}{n}, \end{aligned}$$

and the leakage-inductor current continues to vary linearly according to

5 $$\begin{aligned} \frac{di_{L_k}}{dt}=\frac{-V_P/n + V_S}{L_k} \end{aligned}$$

### Sub-interval-4 $(t_3 \le t < T_S)$

Finally, the secondary bridge changes polarity again, while the primary bridge remains reversed, restoring the voltage difference across the leakage inductance. The corresponding instantaneous voltages are

$$\begin{aligned} v_p=-V_P,\qquad v_s=+V_S,\qquad v_p'=\frac{v_p}{n}, \end{aligned}$$

and the current slope becomes

6 $$\begin{aligned} \frac{di_{L_k}}{dt}=\frac{-V_P/n - V_S}{L_k} \end{aligned}$$

At the end of this interval, $i_{L_k}$ returns to its initial value, thereby completing one switching cycle.

The four sub-intervals show that SPS operation produces a piecewise-linear leakage-inductor current waveform whose slope is determined by the instantaneous difference between the referred primary voltage and the secondary bridge voltage. For the control-oriented averaged model used in Section 4, these switching details are condensed into an equivalent averaged bridge-input quantity $v_{p,\textrm{avg}}$. In the present formulation, $v_{p,\textrm{avg}}$ is not the arithmetic average of the square-wave bridge voltage over a switching cycle; rather, it is a power-equivalent averaged referred-primary input used to preserve the same average power-transfer effect on the leakage branch at the operating point.

A convenient way to define this quantity is through average power over one switching period $T_S$. Let

7 $$\begin{aligned} {\bar{i}}_{L_k}=\frac{1}{T_S}\int _{0}^{T_S} i_{L_k}(t)\,dt \end{aligned}$$

denote the average leakage-branch current. The average power transferred from the referred-primary side into the leakage branch is then

8 $$\begin{aligned} P_{\textrm{DAB}}(\phi )=\frac{1}{T_S}\int _{0}^{T_S} v_p'(t)\,i_{L_k}(t)\,dt \end{aligned}$$

The equivalent averaged input $v_{p,\textrm{avg}}$ is defined so that it produces the same average power when multiplied by the average leakage current, namely

9 $$\begin{aligned} P_{\textrm{DAB}}(\phi )=v_{p,\textrm{avg}}(\phi )\,{\bar{i}}_{L_k} \end{aligned}$$

which gives

10 $$\begin{aligned} v_{p,\textrm{avg}}(\phi )= \frac{1}{T_S\,{\bar{i}}_{L_k}} \int _{0}^{T_S} v_p'(t)\,i_{L_k}(t)\,dt \end{aligned}$$

Equations (3)–(6) determine the piecewise-linear current $i_{L_k}(t)$ used in (10). Under the ideal SPS assumptions adopted here, the same average power is given by the standard SPS power-transfer relation

11 $$\begin{aligned} P_{\textrm{DAB}}=\frac{\eta V_g V_s}{\omega L_k}\,\phi \left( 1-\frac{\phi }{\pi }\right) \end{aligned}$$

so that substitution of (11) into (9) yields the control-oriented equivalent input relation

12 $$\begin{aligned} v_{p,\textrm{avg}}(\phi )= \frac{\eta V_g V_s}{\omega L_k {\bar{i}}_{L_k}}\, \phi \left( 1-\frac{\phi }{\pi }\right) \end{aligned}$$

In the subsequent state-space model, $v_{p,\textrm{avg}}(\phi )$ is therefore used as a control-oriented, power-equivalent input surrogate rather than as the arithmetic average of the switched bridge waveform. Equation (12) makes the functional dependence on phase shift explicit and provides the quantity used for the subsequent local linearization. Thus, the interval analysis supplies the piecewise voltage and current relations, while (10)–(12) convert those switching relations into a power-equivalent averaged input suitable for control-oriented modeling.

Around a nominal operating point ${\bar{\phi }}$, the equivalent averaged input is linearized as

13 $$\begin{aligned} v_{p,\textrm{avg}}(\phi )\approx {\bar{v}}_{p,\textrm{avg}} + k_{\phi }(\phi -{\bar{\phi }}), \qquad k_{\phi }= \left. \frac{\partial v_{p,\textrm{avg}}}{\partial \phi }\right| _{{\bar{\phi }}} \end{aligned}$$

which provides the bridge between the switching-interval description in this section and the small-signal model used for local control analysis in Section 4.

## State-space modeling and small-signal analysis

The cascaded DAB–Čuk converter exhibits coupled dynamics because the DAB-regulated power transfer and the downstream duty-ratio action both affect the intermediate DC-link state. To analyze these interactions in a control-oriented manner, a large-signal averaged model is first developed and then linearized around a steady operating point. The formulation presented in this section is derived under continuous-conduction-mode operation, ideal switching assumptions, and symmetric bridge operation. These assumptions are consistent with the analytical objective of obtaining a tractable model for controller development and interpretation. The use of state-space averaging for control-oriented converter analysis is well established in PWM converter design^18^, while DAB-oriented analytical studies have previously focused mainly on modulation and steady-state behavior^2–4,6^. In the present work, the emphasis is on extending that control-oriented viewpoint to a unified cascaded DAB-based structure with an explicitly modeled DC-link interaction.

### Large-signal averaged modeling

Let the downstream Čuk-stage state vector be defined as

$$\begin{aligned} {\mathbf{x}}_{\textrm{Cuk}}= \begin{bmatrix} i_{L_1}&v_{C_1}&i_{L_2}&v_o \end{bmatrix}^{\top }. \end{aligned}$$

Under CCM and the sign convention adopted in this manuscript, the downstream Čuk converter is described by two switched submodels over one PWM period. When the controlled switch is ON, the corresponding state equations are written as

14 $$\begin{aligned} L_1\frac{di_{L_1}}{dt}&= v_s \end{aligned}$$

15 $$\begin{aligned} C_1\frac{dv_{C_1}}{dt}&= -\,i_{L_2} \end{aligned}$$

16 $$\begin{aligned} L_2\frac{di_{L_2}}{dt}&= v_{C_1}-v_o \end{aligned}$$

17 $$\begin{aligned} C_2\frac{dv_o}{dt}&= i_{L_2}-\frac{v_o}{R_o} \end{aligned}$$

whereas when the controlled switch is OFF, the state equations become

18 $$\begin{aligned} L_1\frac{di_{L_1}}{dt}&= v_s-v_{C_1} \end{aligned}$$

19 $$\begin{aligned} C_1\frac{dv_{C_1}}{dt}&= i_{L_1} \end{aligned}$$

20 $$\begin{aligned} L_2\frac{di_{L_2}}{dt}&= -\,v_o \end{aligned}$$

21 $$\begin{aligned} C_2\frac{dv_o}{dt}&= i_{L_2}-\frac{v_o}{R_o}. \end{aligned}$$

Here $v_{C_1}$ is defined with the polarity seen by the output-inductor branch, and $i_{L_2}$ is taken as positive in the battery-current direction used throughout the manuscript; these sign conventions determine the signs of the coupling terms in the switched and averaged equations.

Using the duty ratio $D_{S1}$, the standard duty-affine averaged switched-network form is

22 $$\begin{aligned} \dot{{\mathbf{x}}}_{\textrm{Cuk}} = D_{S1}\,{\mathbf{f}}_{\textrm{on}}({\mathbf{x}}_{\textrm{Cuk}},v_s) + (1-D_{S1})\,{\mathbf{f}}_{\textrm{off}}({\mathbf{x}}_{\textrm{Cuk}},v_s), \end{aligned}$$

or, equivalently,

23 $$\begin{aligned} \dot{{\mathbf{x}}}_{\textrm{Cuk}} = {\mathbf{f}}_{0}({\mathbf{x}}_{\textrm{Cuk}},v_s) + D_{S1}\,{\mathbf{g}}_{D}({\mathbf{x}}_{\textrm{Cuk}},v_s), \end{aligned}$$

where ${\mathbf{f}}_{0}(\cdot )$ denotes the duty-independent part of the averaged dynamics and ${\mathbf{g}}_{D}(\cdot )$ denotes the duty-dependent contribution. From (14)–(21), the averaged downstream Čuk equations can be written explicitly as

24 $$\begin{aligned} L_1\frac{di_{L_1}}{dt}&= v_s-(1-D_{S1})v_{C_1} \end{aligned}$$

25 $$\begin{aligned} C_1\frac{dv_{C_1}}{dt}&= (1-D_{S1})i_{L_1}-D_{S1}i_{L_2} \end{aligned}$$

26 $$\begin{aligned} L_2\frac{di_{L_2}}{dt}&= D_{S1}v_{C_1}-v_o \end{aligned}$$

27 $$\begin{aligned} C_2\frac{dv_o}{dt}&= i_{L_2}-\frac{v_o}{R_o}. \end{aligned}$$

Under CCM steady-state conditions, setting the derivatives in Eqs. (24)–(27) to zero recovers the voltage-conversion magnitude relation given earlier in Eq. (2), confirming consistency between the switched, averaged, and steady-state descriptions under the adopted polarity convention.

These equations make explicit where the Čuk duty ratio enters the unified model. The corresponding switched derivation is summarized in Appendix A.

Based on Kirchhoff’s laws and the averaged switch representations of the DAB and Čuk stages, the unified nonlinear large-signal equations governing the energy-storage elements are therefore written as

28 $$\begin{aligned} L_k \frac{d i_{L_k}}{dt}&= v_{p,\textrm{avg}}(\phi ) - n v_s \end{aligned}$$

29 $$\begin{aligned} C_s \frac{d v_s}{dt}&= n i_{L_k} - i_{L_1} \end{aligned}$$

30 $$\begin{aligned} L_1 \frac{d i_{L_1}}{dt}&= v_s-(1-D_{S1})v_{C_1} \end{aligned}$$

31 $$\begin{aligned} C_1 \frac{d v_{C_1}}{dt}&= (1-D_{S1})i_{L_1}-D_{S1}i_{L_2} \end{aligned}$$

32 $$\begin{aligned} L_2 \frac{d i_{L_2}}{dt}&= D_{S1}v_{C_1}-v_o \end{aligned}$$

33 $$\begin{aligned} C_2 \frac{d v_o}{dt}&= i_{L_2} - \frac{v_o}{R_o} \end{aligned}$$

Here $L_k$ and $C_s$ correspond to the DAB leakage inductance and the intermediate DC-link capacitor, respectively, while $L_1$, $C_1$, $L_2$, and $C_2$ denote the downstream Čuk-stage elements. The term $v_{p,\textrm{avg}}(\phi )$ represents the equivalent averaged bridge input associated with the DAB stage and is treated as a control-oriented quantity related to the phase-shift angle $\phi$ through (12).

The large-signal equations make the DC-link interaction explicit. In particular, the same intermediate capacitor that buffers energy between the two stages also introduces a dynamic pathway through which DAB and Čuk control actions influence one another. For this reason, the cascaded converter is modeled here as a unified system rather than as two independently analyzed stages.

### Small-signal linearization

To obtain a model suitable for closed-loop analysis, each state variable is decomposed into a steady-state component and a small perturbation component about the nominal operating point as $x={\bar{x}}+\hat{x}$, where $|\hat{x}| \ll |{\bar{x}}|$. From (12), the DAB equivalent averaged-input term is written as

34 $$\begin{aligned} v_{p,\textrm{avg}}(\phi )= \frac{\eta V_g {\bar{v}}_s}{\omega L_k {\bar{i}}_{L_k}}\, \phi \left( 1-\frac{\phi }{\pi }\right) \end{aligned}$$

for the nominal operating condition used in the present local model. Linearizing (34) about ${\bar{\phi }}$ gives

35 $$\begin{aligned} v_{p,\textrm{avg}}= {\bar{v}}_{p,\textrm{avg}} + k_{\phi }{\hat{\phi }}, \qquad k_{\phi }= \left. \frac{\partial v_{p,\textrm{avg}}}{\partial \phi }\right| _{{\bar{\phi }}} = \frac{\eta V_g {\bar{v}}_s}{\omega L_k {\bar{i}}_{L_k}} \left( 1-\frac{2{\bar{\phi }}}{\pi }\right) . \end{aligned}$$

Thus, $k_{\phi }$ is the local slope of the equivalent averaged DAB input with respect to the phase shift at the nominal operating point.

For the downstream Čuk stage, the duty-ratio-dependent perturbation terms follow directly from linearization of (24)–(27) with respect to the state vector and $D_{S1}$. In Jacobian form,

36 $$\begin{aligned} \widehat{\dot{{\mathbf{x}}}}_{\textrm{Cuk}} = {\mathbf{A}}_{\textrm{Cuk}}\hat{{\mathbf{x}}}_{\textrm{Cuk}} + \left. \frac{\partial \dot{{\mathbf{x}}}_{\textrm{Cuk}}}{\partial D_{S1}} \right| _{\bar{{\mathbf{x}}},{\bar{D}}_{S1}} \hat{D}_{S1}, \end{aligned}$$

which gives the duty-ratio perturbation terms in the linearized equations below. Substituting these perturbations into the averaged model and neglecting higher-order terms yields the small-signal equations

37 $$\begin{aligned} \dot{\hat{i}}_{L_k}&= \frac{k_{\phi }}{L_k}{\hat{\phi }} - \frac{n}{L_k}\hat{v}_s \end{aligned}$$

38 $$\begin{aligned} \dot{\hat{v}}_s&= \frac{n}{C_s}\hat{i}_{L_k} - \frac{1}{C_s}\hat{i}_{L_1} \end{aligned}$$

39 $$\begin{aligned} \dot{\hat{i}}_{L_1}&= \frac{1}{L_1}\hat{v}_s - \frac{1-{\bar{D}}_{S1}}{L_1}\hat{v}_{C_1} + \frac{{\bar{v}}_{C_1}}{L_1}\hat{D}_{S1} \end{aligned}$$

40 $$\begin{aligned} \dot{\hat{v}}_{C_1}&= \frac{1-{\bar{D}}_{S1}}{C_1}\hat{i}_{L_1} - \frac{{\bar{D}}_{S1}}{C_1}\hat{i}_{L_2} - \frac{{\bar{i}}_{L_1}+{\bar{i}}_{L_2}}{C_1}\hat{D}_{S1} \end{aligned}$$

41 $$\begin{aligned} \dot{\hat{i}}_{L_2}&= \frac{{\bar{D}}_{S1}}{L_2}\hat{v}_{C_1} - \frac{1}{L_2}\hat{v}_o + \frac{{\bar{v}}_{C_1}}{L_2}\hat{D}_{S1} \end{aligned}$$

42 $$\begin{aligned} \dot{\hat{v}}_o&= \frac{1}{C_2}\hat{i}_{L_2} - \frac{1}{R_o C_2}\hat{v}_o \end{aligned}$$

The resulting linearized system shows that perturbations in both the DAB phase shift ${\hat{\phi }}$ and the Čuk duty ratio $\hat{D}_{S1}$ propagate through the intermediate DC-link node $\hat{v}_s$. The finite value of the DC-link capacitor therefore prevents the two stages from being dynamically independent, even though the controller is organized hierarchically. This observation motivates the use of a hierarchical control structure rather than interpreting the system as perfectly decoupled over the full operating range. The present small-signal model is derived under CCM, ideal switching, and nominal operating-point assumptions and is therefore intended for local control analysis rather than wide-range nonlinear prediction.

The nominal operating point used for local linearization and controller interpretation is summarized in Table 3. The gain $k_{\phi }$ was evaluated from (35) at this operating point, and the resulting small-signal model is therefore intended for local control-oriented interpretation around the reported G2V operating condition.

**Table 3:** Nominal operating point used for small-signal linearization.

Variable	Symbol	Nominal value	Units	How obtained
Leakage current	${\bar{i}}_{L_k}$	6.5	A	Averaged simulation
DC-link voltage	${\bar{v}}_s$	30	V	Selected nominal G2V operating point, corroborated by experiment
Čuk input inductor current	${\bar{i}}_{L_1}$	6.0	A	Simulation
Energy-transfer capacitor voltage	${\bar{v}}_{C_1}$	15	V	Simulation
Čuk output inductor current	${\bar{i}}_{L_2}$	20	A	Simulation
Output voltage	${\bar{v}}_o$	24	V	Design target
DAB phase shift	${\bar{\phi }}$	$18^\circ$	deg	Controller setting
Čuk duty ratio	${\bar{D}}_{S1}$	0.45	–	Controller setting
Input voltage	$V_g$	60	V	Fixed
Load resistance	$R_o$	1.2	$\Omega$	Equivalent battery load
DAB averaged bridge voltage	${\bar{v}}_{p,\textrm{avg}}$	32	V	Evaluated from (34) at the nominal point
Linearization gain	$k_\phi$	85	V/rad	Evaluated from (35) at the nominal point

### State-space representation and control interpretation

Defining the state vector and the control-input vector as

43 $$\begin{aligned} \hat{{\mathbf{x}}}= \begin{bmatrix} \hat{i}_{L_k}&\hat{v}_s&\hat{i}_{L_1}&\hat{v}_{C_1}&\hat{i}_{L_2}&\hat{v}_o \end{bmatrix}^{\top }, \qquad \hat{{\mathbf{u}}}= \begin{bmatrix} {\hat{\phi }}&\hat{D}_{S1} \end{bmatrix}^{\top } \end{aligned}$$

the linearized dynamics can be written in compact state-space form as

44 $$\begin{aligned} \dot{\hat{{\mathbf{x}}}}={\mathbf{A}}\hat{{\mathbf{x}}}+{\mathbf{B}}\hat{{\mathbf{u}}}, \qquad \hat{{\mathbf{y}}}={\mathbf{C}}\hat{{\mathbf{x}}} \end{aligned}$$

where, consistent with the outer-loop control objective described in Section 2, the model output is taken as the Čuk output-inductor current,

45 $$\begin{aligned} \hat{{\mathbf{y}}}=\hat{i}_{L_2}. \end{aligned}$$

Under the CCM operating condition considered here, $\hat{i}_{L_2}$ is the model variable corresponding to the regulated battery current $\hat{I}_o$.

The state, input, and output matrices are

46 $$\begin{aligned} & {\mathbf{A}}= \begin{bmatrix} 0 & -\dfrac{n}{L_k} & 0 & 0 & 0 & 0\\ \dfrac{n}{C_s} & 0 & -\dfrac{1}{C_s} & 0 & 0 & 0\\ 0 & \dfrac{1}{L_1} & 0 & -\dfrac{1-{\bar{D}}_{S1}}{L_1} & 0 & 0\\ 0 & 0 & \dfrac{1-{\bar{D}}_{S1}}{C_1} & 0 & -\dfrac{{\bar{D}}_{S1}}{C_1} & 0\\ 0 & 0 & 0 & \dfrac{{\bar{D}}_{S1}}{L_2} & 0 & -\dfrac{1}{L_2}\\ 0 & 0 & 0 & 0 & \dfrac{1}{C_2} & -\dfrac{1}{R_o C_2} \end{bmatrix}, \end{aligned}$$

47 $$\begin{aligned} & {\mathbf{B}}= \begin{bmatrix} \dfrac{k_{\phi }}{L_k} & 0\\ 0 & 0\\ begin{eqnarray*}6pt] 0 & \dfrac{{\bar{v}}_{C_1}}{L_1}\\ 0 & -\dfrac{{\bar{i}}_{L_1}+{\bar{i}}_{L_2}}{C_1}\\ 0 & \dfrac{{\bar{v}}_{C_1}}{L_2}\\ 0 & 0 \end{bmatrix}, \qquad {\mathbf{C}}= \begin{bmatrix} 0&0&0&0&1&0 \end{bmatrix}. \end{aligned}$$

Equations (44)–(47) constitute the unified small-signal model of the cascaded DAB–Čuk converter. Let ${\mathbf{b}}_1$ and ${\mathbf{b}}_2$ denote the first and second columns of ${\mathbf{B}}$, respectively. The first corresponds to the DAB phase-shift input ${\hat{\phi }}$, while the second corresponds to the Čuk duty-ratio input $\hat{D}_{S1}$. The corresponding local control-to-output transfer functions are

48 $$\begin{aligned} G_{i_{L_2}\phi }(s)={\mathbf{C}}(s{\mathbf{I}}-{\mathbf{A}})^{-1}{\mathbf{b}}_1, \qquad G_{i_{L_2}D}(s)={\mathbf{C}}(s{\mathbf{I}}-{\mathbf{A}})^{-1}{\mathbf{b}}_2 . \end{aligned}$$

These transfer functions are used for local controller interpretation because $i_{L_2}$ represents the regulated battery current $I_o$ under the CCM operating condition adopted in this work.

**Fig. 5:**
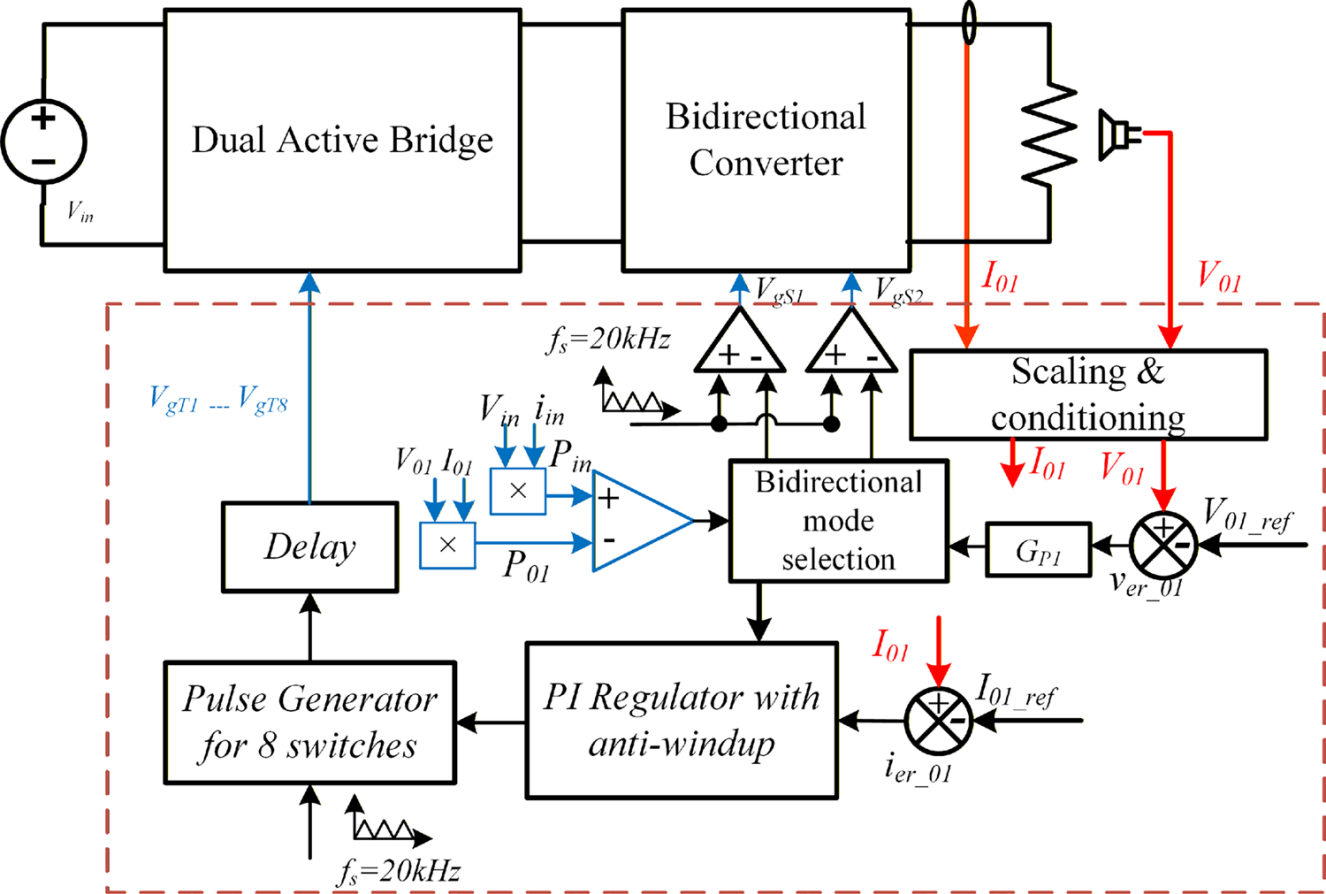
Hierarchical dual-loop control structure used for the bidirectional DAB–Čuk converter, where the inner loop regulates the intermediate DC-link voltage $V_s$ through DAB phase shift and the outer loop regulates the battery current $I_o$ through Čuk duty ratio.

Figure 5 summarizes the controller structure used in this work. Two PI controllers are employed in a hierarchical dual-loop arrangement. The inner loop acts on the DAB phase shift to regulate the intermediate DC-link voltage $V_s$ and is designed to respond faster than the outer loop. The outer loop acts on the Čuk duty ratio to regulate the battery current $I_o$, represented in the model by $i_{L_2}$. In the present study, the controller gains were selected from the nominal small-signal transfer functions in (48) so as to obtain stable regulation with clear bandwidth separation between the fast inner loop and the slower outer loop. The inner-loop PI gains were tuned first around the nominal operating point, after which the outer-loop PI gains were selected on the basis of the resulting slower battery-current plant. The bandwidth and phase-margin values reported in Table 4 were obtained from the nominal linearized model and then checked in simulation prior to experimental implementation. In the simulation study, settling time was evaluated as the time required for the controlled variable to enter and remain within a $\pm 5\%$ band around its final value, and overshoot was computed relative to the corresponding final command value.

**Table 4:** Controller implementation and local design basis.

Loop	Ctrl. var.	Manip. var.	Type	$\mathbf {K_p}$	$\mathbf {K_i}$	Rate	PWM	Limits	Anti-windup	Filter	PM
Inner	$V_s$	$\phi$	PI	0.8	120	40 kHz	$0.5^\circ$	$\pm 30^\circ$	Back-calc.	LPF, 2 kHz; BW $=1.5$ kHz	$55^\circ$
Outer	$I_o$	$D_{S1}$	PI	0.5	60	20 kHz	10-bit	0–0.8	Clamping	LPF, 500 Hz; BW $=300$ Hz	$50^\circ$

This state-space representation is therefore used in a control-oriented sense: it captures the dominant coupling between the two stages, supports interpretation of the hierarchical control structure, and provides a basis for understanding the closed-loop behavior reported in Sections 5 and 6.

### Local stability analysis of the hierarchical control system

**Fig. 6:**
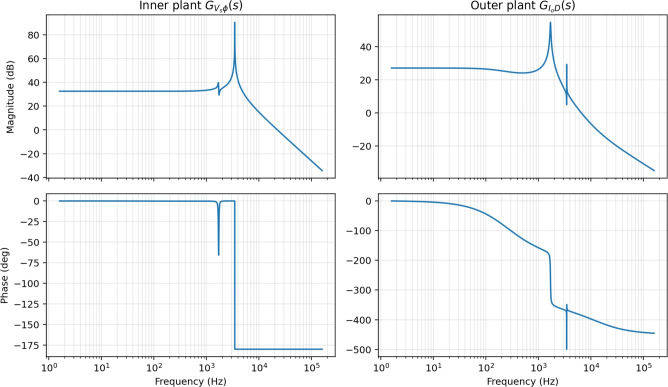
Nominal Bode plots of the two plant channels used for hierarchical control interpretation: (**a**) and (**b**) the inner-loop plant $G_{V_s\phi }(s)$, and (**c**) and (**d**) the outer-loop plant $G_{I_oD}(s)$, evaluated from the linearized model at the nominal operating point.

For stability discussion, it is useful to distinguish the two nominal plant channels that are directly used in the hierarchical design, namely the DC-link channel from DAB phase shift to intermediate voltage and the battery-current channel from downstream duty ratio to output-inductor current,

49 $$\begin{aligned} G_{V_s\phi }(s)={\mathbf{C}}_{V_s}(s{\mathbf{I}}-{\mathbf{A}})^{-1}{\mathbf{b}}_1, \qquad G_{I_oD}(s)={\mathbf{C}}_{I}(s{\mathbf{I}}-{\mathbf{A}})^{-1}{\mathbf{b}}_2 \end{aligned}$$

where ${\mathbf{C}}_{V_s}=[0\;1\;0\;0\;0\;0]$ and ${\mathbf{C}}_{I}=[0\;0\;0\;0\;1\;0]$. The first channel is used for the inner DC-link loop and the second for the outer battery-current loop. Figure 6 shows the nominal Bode plots of these two plant channels evaluated from the linearized model at the operating point of Table 3. The $V_s$–$\phi$ channel is the faster channel around the nominal point, which supports its use as the inner loop, whereas the battery-current channel is deliberately regulated as the slower outer loop.

The nominal eigenvalues of the open-loop small-signal state matrix ${\mathbf{A}}$, evaluated with the component values of Table 9 and the nominal point of Table 3, are listed in Table 5. All open-loop eigenvalues remain in the left-half plane about the selected operating point, indicating that the averaged plant is internally stable prior to controller closure. The two dominant oscillatory pairs correspond to the coupled DAB/DC-link dynamics and the downstream energy-transfer dynamics, while the remaining real poles are appreciably faster and more strongly damped.

**Table 5:** Nominal open-loop eigenvalues of the linearized small-signal plant.

Mode	Eigenvalue	Interpretation
1	$-0.155 \pm j\,21718.243$	Fast oscillatory DAB/DC-link pair
2	$-127.096 \pm j\,10633.929$	Coupled downstream oscillatory pair
3	$-1220.639$	Damped real mode
4	$-16255.355$	Fast damped real mode

The controller settings in Table 4 were then selected so that the inner $V_s$-loop retains a nominal bandwidth of approximately $1.5~\textrm{kHz}$ with a reported phase margin of $55^{\circ }$, while the outer $I_o$-loop retains a nominal bandwidth of approximately $300~\textrm{Hz}$ with a reported phase margin of $50^{\circ }$. The resulting bandwidth ratio is therefore about $5{:}1$, which is sufficient for the present hierarchical implementation because the faster DAB loop regulates the intermediate energy buffer before the slower battery-current loop acts on the downstream stage. In this sense, the present controller is not assumed to be perfectly decoupled; rather, it is organized so that the dominant interstage interaction is managed through bandwidth separation.

To check whether the local plant description is unduly fragile to moderate parameter uncertainty, a simple sensitivity study was also carried out by perturbing four representative parameters by $\pm 20\%$: the DAB leakage inductance $L_k$, the DC-link capacitance $C_s$, the equivalent battery/load resistance $R_o$, and one downstream Čuk energy-storage element $C_1$. Table 6 reports the maximum real part among the perturbed open-loop eigenvalues for each case. In all tested perturbations, the maximum real part remained negative, which indicates that the nominal local plant remained internally stable under these moderate variations; the closest approach to the imaginary axis occurred for the $-20\%$ perturbation of $L_k$ and $C_s$, but the poles still remained in the left-half plane.

**Table 6:** Open-loop eigenvalue sensitivity of the nominal small-signal plant under representative $\pm 20\%$ parameter perturbations.

Perturbation	Variation	Max. real part	Comment
$L_k$	$-20\%$	$-0.063$	Dominant pair moves closer to the imaginary axis but remains stable.
$L_k$	$+20\%$	$-0.327$	Damping of the dominant oscillatory pair increases.
$C_s$	$-20\%$	$-0.076$	DC-link mode becomes less damped, but remains in the left-half plane.
$C_s$	$+20\%$	$-0.282$	DC-link mode remains stable with modest additional damping.
$R_o$	$-20\%$	$-0.156$	Load variation has limited influence on the dominant real part.
$R_o$	$+20\%$	$-0.149$	Plant remains locally stable.
$C_1$	$-20\%$	$-0.282$	Downstream oscillatory pair remains adequately damped.
$C_1$	$+20\%$	$-0.098$	Reduced damping but no pole migration to the right-half plane.

The above results do not establish global stability over the entire operating envelope, nor do they replace broader nonlinear validation. However, they do provide an explicit local stability justification for the small-signal design basis used in this paper: the nominal plant is internally stable about the selected operating point, the inner and outer plants are sufficiently separated in speed to support hierarchical control, the adopted PI gains retain the reported local phase-margin and bandwidth targets of Table 4, and moderate representative parameter perturbations do not destabilize the open-loop plant.

## Simulation results

The proposed cascaded DAB–Čuk converter was evaluated in MATLAB/Simulink under both open-loop and closed-loop conditions. The aim of the simulation study is to examine whether the unified modeling framework and the hierarchical control structure are consistent with the expected operating behavior of a DAB-based bidirectional charging interface^2,9–11^. The simulated control arrangement follows Fig. 5, in which the inner loop acts on the DAB phase shift to regulate the intermediate DC-link variable and the outer loop acts on the downstream duty ratio to regulate the battery-side current. The principal simulation conditions are summarized in Table 7.

**Table 7:** Simulation conditions used in the MATLAB/Simulink study.

Parameter	Value
Input voltage $V_g$	$60~\textrm{V}$
Switching frequency $f_s$	$20~\textrm{kHz}$
Transformer turns ratio $n$	$2{:}1$
Intermediate DC-link capacitance $C_s$	$220~\mu \textrm{F}$
Controller type	Hierarchical PI (inner loop regulates $V_s$, outer loop regulates $I_o$)
Battery model	24 V battery model, initial terminal voltage $23.5~\textrm{V}$, initial SOC $40\%$

### Open-loop operation

**Fig. 7:**
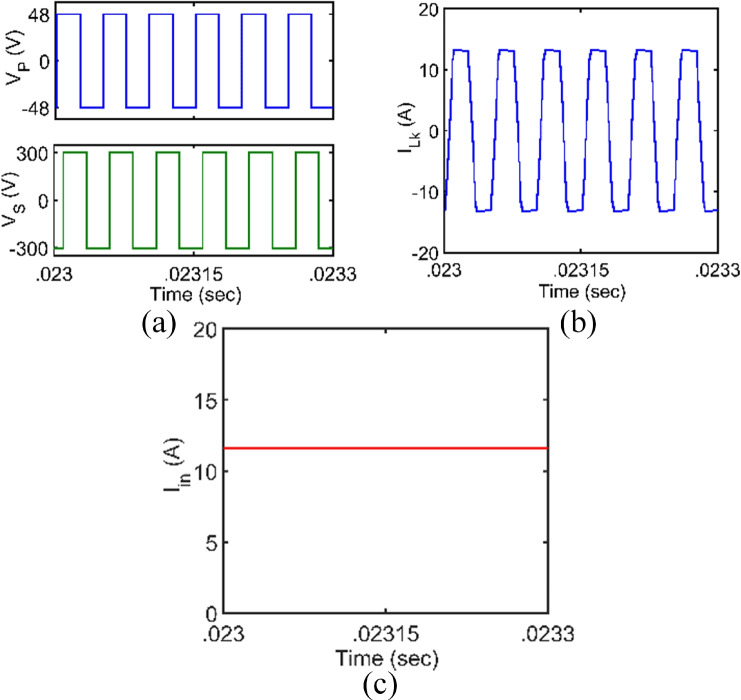
Open-loop simulation results for $\phi /\pi =0.33$ and $R_o=220~\Omega$: (**a**) primary and secondary bridge voltages, (**b**) leakage-inductor current, and (**c**) input current, showing low transferred-power operation under SPS control.

**Fig. 8:**
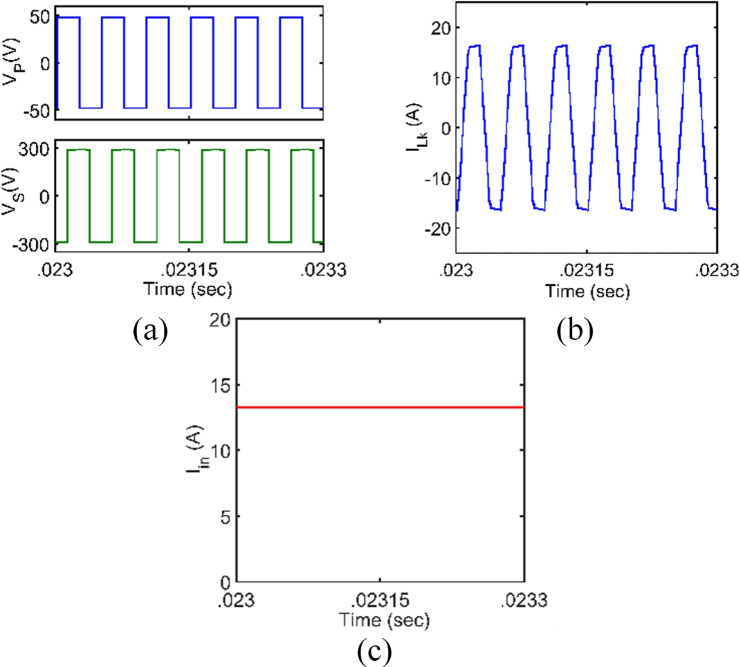
Open-loop simulation results for $\phi /\pi =0.50$ and $R_o=220~\Omega$: (**a**) primary and secondary bridge voltages, (**b**) leakage-inductor current, and (**c**) input current, showing increased transferred-power operation compared with Fig. 7.

In the open-loop study, the DAB stage was simulated for multiple normalized phase-shift conditions in order to examine the expected dependence of transferred power on SPS control. For the first case, the normalized phase shift was set to $\phi /\pi =0.33$ with a resistive load of $R_o=220~\Omega$. The corresponding primary and secondary bridge voltages, transformer leakage-inductor current, and input current are shown in Fig. 7. For the second case, the normalized phase shift was increased to $\phi /\pi =0.50$ while maintaining the same load condition, and the corresponding waveforms are shown in Fig. 8.

In both cases, the primary and secondary bridge voltages exhibit the expected SPS-imposed phase displacement, and the leakage-inductor current shows the piecewise-linear waveform associated with the interval analysis of Section 3. At the lower normalized phase shift, the current amplitude is smaller, indicating a lower transferred-power condition. As the phase shift increases, the inductor-current amplitude also increases, indicating a higher transferred-power condition. This trend is consistent with the standard SPS power-transfer characteristic of the DAB stage,

50 $$\begin{aligned} P_{\textrm{DAB}} \propto \phi \left( 1-\frac{\phi }{\pi }\right) . \end{aligned}$$

The input-current waveforms remain continuous under the simulated conditions, which is also consistent with the downstream filtering behavior introduced by the cascaded topology. These open-loop results should therefore be interpreted as physics-consistency checks of the expected SPS operating trends rather than as quantitative validation of the full converter model. Quantitatively, increasing the phase shift from $\phi /\pi =0.33$ to $\phi /\pi =0.50$ increased the leakage-current peak from $6.5~\textrm{A}$ to $9.2~\textrm{A}$ and the estimated input power from $348~\textrm{W}$ to $366~\textrm{W}$, which is consistent with the expected SPS power-transfer trend.

### Closed-loop battery-charging operation

The cascaded converter was also simulated under battery-charging conditions using the dual-loop controller described in Section 4. In this case, the DAB stage performs isolation and intermediate energy regulation, while the downstream Čuk converter regulates the battery-side current during the charging process.

**Fig. 9:**
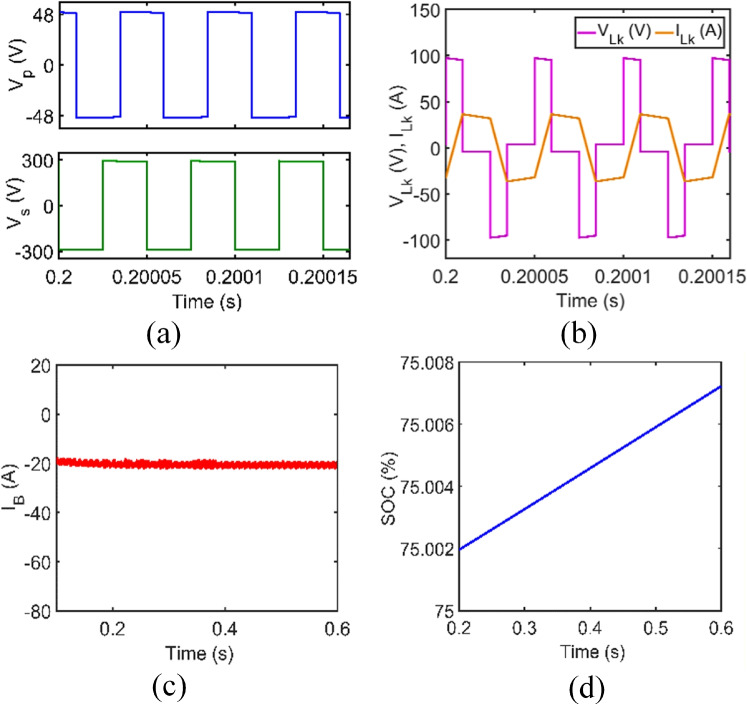
Closed-loop simulation results for battery charging under SPS control in the constant-current interval: (**a**) primary and secondary bridge voltages, (**b**) inductor voltage and current, (**c**) battery current, and (**d**) state of charge.

During this operation, the inner phase-shift control loop regulates the intermediate DC-link variable to support stable power transfer from the source side toward the battery side. The outer loop adjusts the downstream duty ratio in accordance with the current-regulation requirement. For the reported closed-loop simulation case, the DAB phase shift remained within the implemented controller range, with a nominal operating value of $18^\circ$, thereby maintaining consistency with the experimental controller saturation limits. Figure 9a shows that the primary and secondary bridge voltages remain phase shifted under closed-loop operation, indicating that SPS modulation continues to regulate the DAB power-transfer process. Figure 9b shows the associated inductor voltage and current waveforms, which remain consistent with controlled energy transfer through the leakage path. The battery current in Fig. 9c follows the reported charging command and reaches approximately $20.8~\textrm{A}$, while Fig. 9d shows the corresponding increase in battery state of charge during the charging interval. Under the reported simulation scenario, the charging process proceeds smoothly during the constant-current interval. Because this charging case corresponds to a specific simulated operating point, it should be interpreted separately from the laboratory operating conditions reported in Section 6 unless the same voltage, load, and battery conditions are explicitly matched.

To provide an explicit local transient comparison between the linearized model and the nonlinear simulation, a step in battery-current reference from $10~\textrm{A}$ to $20~\textrm{A}$ was applied at $t=5~\textrm{ms}$ around the nominal operating point. Both the linearized and nonlinear models were evaluated using the same PI gains and the same nominal operating point to ensure consistency in comparison. Settling time was evaluated as the time required for $I_o$ to enter and remain within a $\pm 5\%$ band around the final value, and overshoot was computed relative to the final current command. For this case, the linear small-signal model predicted a settling time of $8.5~\textrm{ms}$, an overshoot of $4.0\%$, and a final current of $20.0~\textrm{A}$, whereas the nonlinear Simulink model gave a settling time of $10.2~\textrm{ms}$, an overshoot of $6.5\%$, and a final current of $20.1~\textrm{A}$. The linear model therefore captures the dominant response trend and final value with modest underprediction of settling time and overshoot, which is consistent with its intended use for local control analysis around the nominal operating point rather than full nonlinear switching-level prediction.

For the reported nonlinear Simulink case, the extracted transient metrics are summarized in Table 8. Together with the corresponding linear-model values, these results indicate local transient agreement around the nominal operating point. Accordingly, the present simulation section should be interpreted as showing local operating-point consistency between the control-oriented model and the nonlinear simulation rather than as a complete quantitative proof of model accuracy over all operating conditions.

Taken together, the simulation results indicate that the hierarchical control structure maintains stable operation of the cascaded converter under the tested charging condition. For the closed-loop G2V case, the converter regulated the DC-link and battery-side variables at approximately $30.0~\textrm{V}$ and $24.1~\textrm{V}$, respectively, with a simulated battery current of $20.8~\textrm{A}$, $10.2~\textrm{ms}$ settling time, and $6.5\%$ overshoot. These results are consistent with the operating trends predicted by the analytical framework, but they should be interpreted as local operating-point results rather than as wide-range validation of the converter model.

**Table 8:** Local transient comparison between the linear small-signal and nonlinear Simulink models for a $10~\textrm{A}\rightarrow 20~\textrm{A}$ battery-current reference step applied at $t=5~\textrm{ms}$.

Model	Settling (ms)	Overshoot (%)	Final (A)	Interpretation
Linear small-signal	8.5	4.0	20.0	Local analytical prediction
Nonlinear Simulink	10.2	6.5	20.1	Switching-model simulation

## Experimental validation

**Fig. 10:**
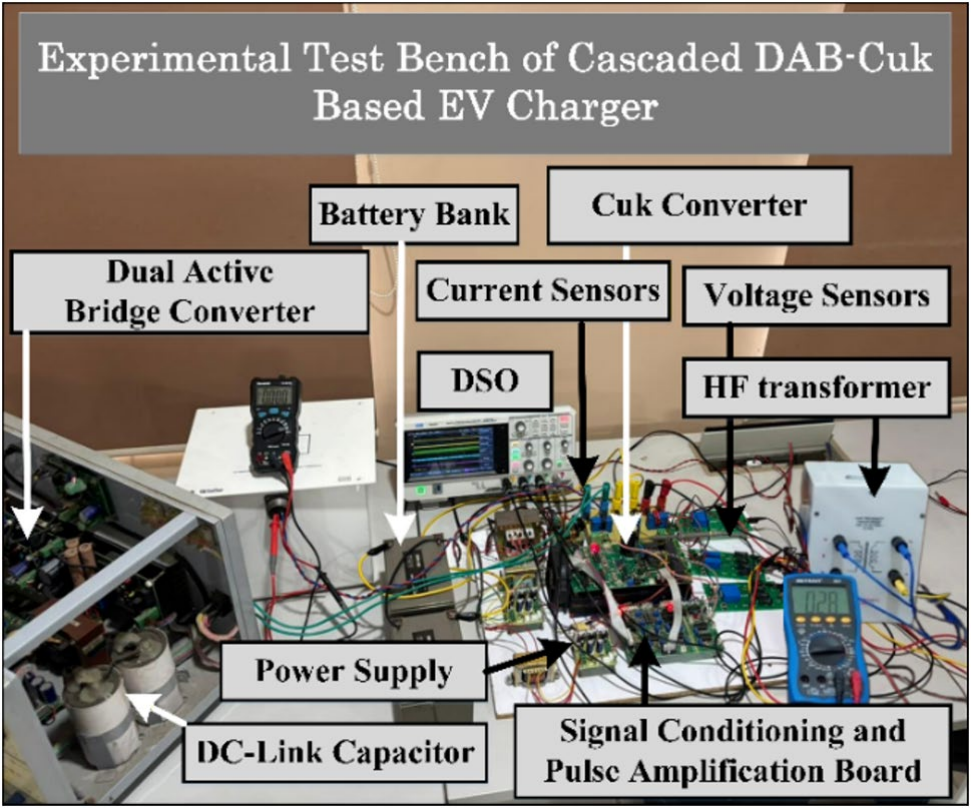
Experimental setup used for laboratory-scale validation of the proposed DAB–Čuk converter and its hierarchical control implementation.

Experimental validation was carried out on a laboratory-scale hardware prototype in order to assess the feasibility of the proposed cascaded DAB–Čuk converter and its hierarchical control structure. The setup shown in Fig. 10 includes the DAB stage with a high-frequency transformer, the downstream Čuk converter, the associated gate-drive and sensing circuits, and a digital controller implementing SPS and duty-ratio control. The principal hardware parameters used in the reported tests are summarized in Table 9. Unless otherwise stated, the experimental waveforms correspond to the laboratory prototype operating at $60~\textrm{V}$ input, $20~\textrm{kHz}$ switching frequency, and the component values listed in Table 9. The present validation is intended as a laboratory-scale demonstration of bidirectional operation in a DAB-based charging interface, consistent with the broader application interest reported in prior DAB charging studies^9–12^.

**Table 9:** Experimental setup parameter specifications.

Parameter	Value
Input voltage	$60~\textrm{V}$
Power MOSFETs	IMW40R045M2HXKSA1
DAB leakage inductance $L_k$	$40~\mu \textrm{H}$
Čuk inductances $(L_1, L_2)$	$333~\mu \textrm{H},\,830~\mu \textrm{H}$
Capacitances $(C_1, C_2)$	$10~\mu \textrm{F},\,47~\mu \textrm{F}$
Intermediate DC-link capacitance $C_s$	$220~\mu \textrm{F}$ low-ESR electrolytic capacitor
Battery configuration	24 V nominal battery pack (two 12 V lead-acid batteries in series)
Battery capacity	$14~\textrm{Ah}$
Test condition	Open-loop resistive-load SPS test only: $R_o=220~\Omega$; closed-loop battery charging/discharging with 24 V, 14 Ah battery pack
Nominal output voltage $V_o$	$24~\textrm{V}$
Digital platform	STM32F4-Discovery board
Operating frequency of DAB and DC–DC converter	$20~\textrm{kHz}$
High-frequency transformer	EE-40 ferrite core, turns ratio $2{:}1$

The instrumentation and test protocol used for the reported measurements are summarized in Tables 10 and 11, respectively.

**Table 10:** Instrumentation and sensing details.

Item	Model/type	Range	Accuracy	Bandwidth	Calibration	Used for
Oscilloscope	Tektronix MDO3024	200 MHz	±1%	200 MHz	Factory + probe check	Waveforms
Voltage probe 1	Tektronix TPP0200	200 V	±2%	200 MHz	Calibrated	$V_p$
Voltage probe 2	Tektronix TPP0100	100 V	±2%	100 MHz	Calibrated	$V_s$
Current probe 1	Tektronix TCPA300 + TCP312	30 A	±3%	100 MHz	Zeroed before run	$i_{L_k}$
Current probe 2	Tektronix TCP202	15 A	±3%	50 MHz	Zeroed before run	$I_{in}$
Battery current sensor	LEM LA 55-P	50 A	±1%	100 kHz	Calibrated with shunt	$I_o$
ADC (controller)	STM32F4 12-bit ADC	0–3.3 V	±1 LSB	1 MSPS	Internal reference	Closed-loop sensing
Signal conditioning	Op-amp scaling + RC filter	–	–	10 kHz	Bench calibrated	Scaling/filtering

**Table 11:** Experimental test protocol.

Test ID	Test article/mode	Initial condition	Runs	Duration	Criterion and purpose
EXP-1	Open-loop SPS validation	No battery; ambient temperature $27^\circ \textrm{C}$	3	0.05 s	Waveform consistency; SPS steady-state validation
EXP-2	Closed-loop G2V with 24 V, 14 Ah battery	Initial battery voltage $23.8~\textrm{V}$, initial SOC $45\%$, ambient temperature $27^\circ \textrm{C}$, rest time 10 min, no preconditioning	3	600 s	$|I_o-I_o^*|<5\%$, no instability; G2V current control
EXP-3	Closed-loop V2G with 24 V, 14 Ah battery	Initial battery voltage $24.2~\textrm{V}$, initial SOC $55\%$, ambient temperature $27^\circ \textrm{C}$, rest time 10 min, no preconditioning	3	600 s	$|I_o-I_o^*|<5\%$, no instability; V2G current control
EXP-4	Bidirectional reversal with 24 V, 14 Ah battery	Initial battery voltage $24.0~\textrm{V}$, initial SOC $50\%$, ambient temperature $27^\circ \textrm{C}$, rest time 10 min, no preconditioning	3	2.0 s	Successful mode reversal with no sustained oscillation; G2V$\leftrightarrow$V2G transient

It should be noted that the present validation is performed on a laboratory-scale prototype under the reported operating conditions. The purpose of the experimental section is therefore to examine hardware feasibility, operating-point current regulation, and bidirectional power-flow reversal under the reported laboratory conditions, rather than to claim full-scale charger qualification or complete dynamic characterization.

### Steady-state SPS operation

**Fig. 11:**
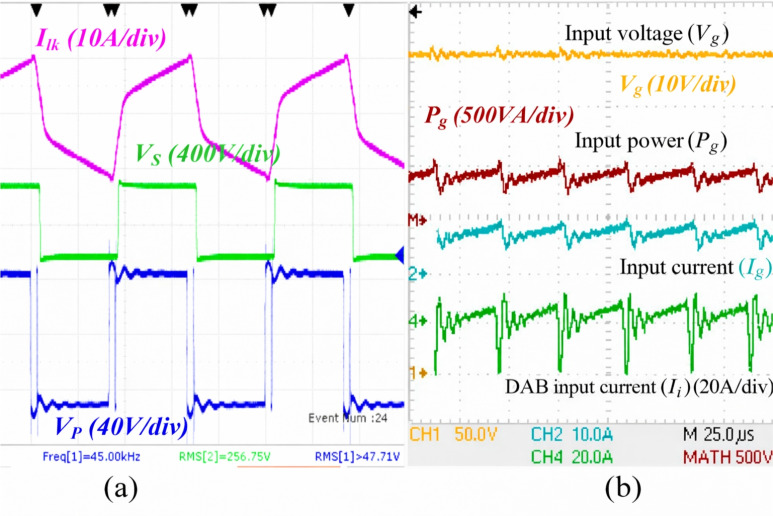
Experimental steady-state SPS waveforms of the cascaded DAB–Čuk converter at a $20^\circ$ phase shift and $20~\textrm{kHz}$ switching frequency: (**a**) primary and secondary bridge voltages together with the leakage current, showing the expected SPS phase displacement and quasi-triangular current shape; (**b**) input voltage $V_g$, source-side input power $P_g$, source-side input current $I_g$, and DAB input current $I_i$, showing sustained forward power transfer under the reported steady-state condition.

Figure 11a shows the measured leakage current together with the primary and secondary bridge voltages under SPS operation at the reported phase shift. The bridge voltages exhibit the expected square-wave behavior with a clear phase displacement, and the measured leakage current shows the quasi-triangular shape associated with controlled energy transfer through the transformer leakage inductance. These waveforms therefore provide a hardware check of the expected SPS waveform shape and phase-displacement relationship. Figure 11b presents the corresponding input-side waveforms. Under the reported test condition, the input current and power traces indicate sustained power transfer without obvious waveform distortion. Taken together, these observations are consistent with stable steady-state SPS operation of the cascaded converter under the reported laboratory condition. For the representative SPS steady-state test, the measured DC-link voltage was approximately $30.0~\textrm{V}$ and the leakage-current peak was approximately $8.7~\textrm{A}$, consistent with the reported forward power-transfer condition.

### Closed-loop G2V and V2G operation

**Fig. 12:**
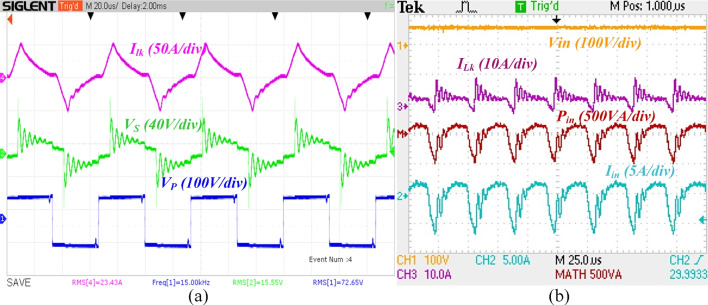
Experimental closed-loop G2V operation of the cascaded DAB–Čuk converter under the reported laboratory operating condition: (**a**) primary and secondary bridge voltages together with the leakage current, showing SPS-mediated forward power transfer; (**b**) input current, input voltage, and input power, corresponding to the nominal G2V operating point for which $I_o\approx 20~\textrm{A}$ and $V_s\approx 29.5~\textrm{V}$.

Closed-loop operation during source-to-battery transfer is illustrated in Fig. 12. The measured waveforms show maintained SPS action in the DAB stage and regulated downstream operation during charging. The bridge-voltage waveforms remain balanced, and the leakage-current shape remains consistent with forward power transfer through the transformer path. Under this reported operating point, the extracted steady-state values were $V_s=29.5~\textrm{V}$, $V_o=23.8~\textrm{V}$, $I_{Lk,pk}=8.5~\textrm{A}$, $I_{in,\textrm{avg}}=8.6~\textrm{A}$, and $I_o=20.0~\textrm{A}$. Accordingly, Fig. 12 should be interpreted as hardware evidence of current regulation at one nominal G2V operating point together with stable SPS-mediated power transfer.

**Fig. 13:**
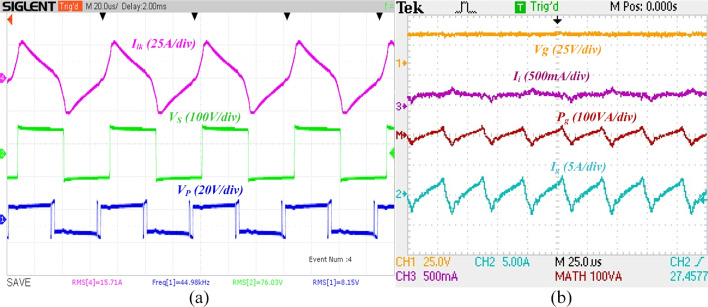
Experimental closed-loop V2G operation of the cascaded DAB–Čuk converter under the reported laboratory operating condition: (**a**) primary and secondary bridge voltages together with the leakage current, showing continued SPS operation under reverse power flow; (**b**) input current, input voltage, and input power, corresponding to the reported reverse-power operating point for which $I_o\approx -15.2~\textrm{A}$ and $V_s$ remained close to $30~\textrm{V}$.

Figure 13 illustrates the reverse operating mode, in which the commanded phase-shift direction is reversed and power is transferred from the battery side back to the DC bus. The reversal of battery-side current confirms bidirectional operation of the cascaded system, while the maintained square-wave bridge voltages and quasi-triangular leakage-current waveform indicate that the DAB stage continues to operate in the intended SPS regime. For the reported V2G case, the measured battery-side current reversed to approximately $-15.2~\textrm{A}$ while the DC-link voltage remained at approximately $30.2~\textrm{V}$, indicating that bidirectional power-flow reversal was achieved without loss of the reported DC-link operating level. This figure should therefore be interpreted as hardware evidence of reverse-power operation at one reported V2G operating point rather than as a complete dynamic characterization over the full operating range.

### Bidirectional operation summary

**Fig. 14:**
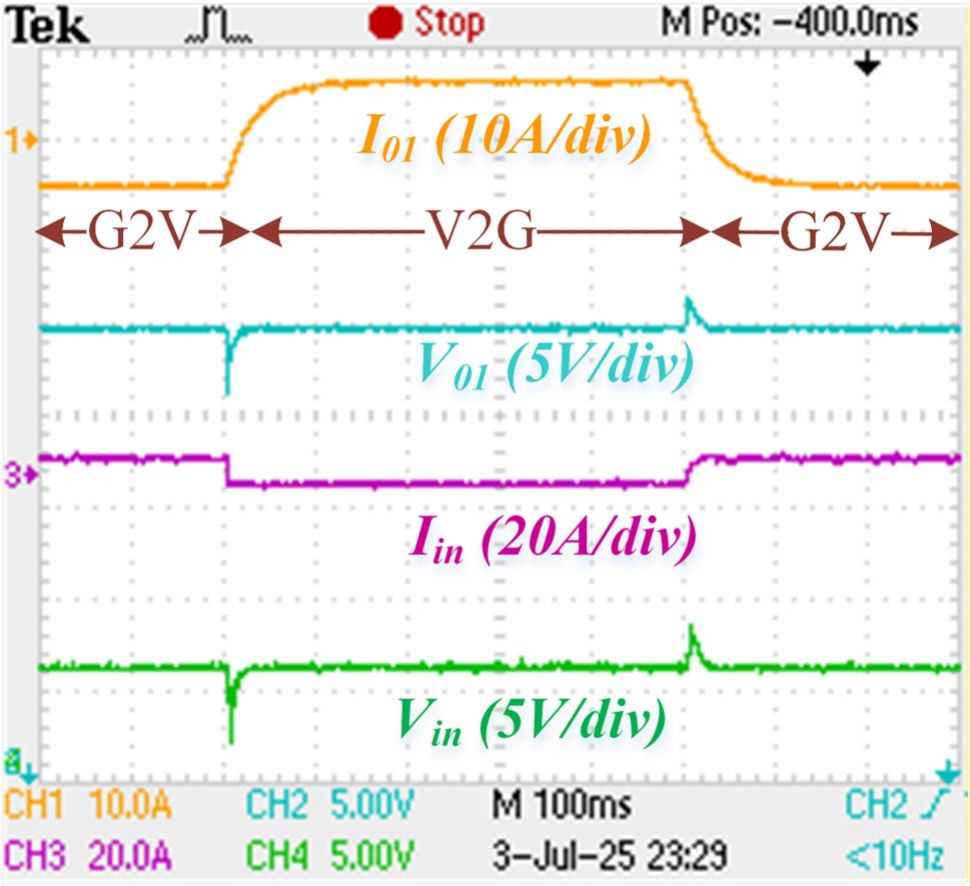
Experimental battery-side response of the cascaded DAB–Čuk converter during the reported G2V$\rightarrow$V2G transition, showing the measured battery voltage $V_o$ and battery current $I_o$. The battery-side variables remain bounded during charging and discharging, current polarity reversal is achieved without sustained oscillation, and the quantified transient metrics are summarized in Table 12.

Figure 14 provides an overall experimental illustration of bidirectional operation between the DC source and the battery side. Under the tested laboratory conditions, the measured battery-side voltage and current remain controlled during both charging and discharging intervals, and reversal of current polarity is achieved during the reported G2V$\leftrightarrow$V2G transition. In conjunction with the EXP-4 test criterion in Table 11, the reported transient case provides qualitative evidence of successful operating-mode reversal without sustained oscillation over the measured interval. This should be interpreted as local transient hardware evidence under the reported prototype condition, not as a full dynamic identification of the converter or as a complete verification of the small-signal model over a broad operating range.

To quantify the reported EXP-4 reversal, the G2V$\rightarrow$V2G transition was evaluated in terms of battery-current settling, current undershoot, and DC-link excursion. Settling time was evaluated as the time required for $I_o$ to enter and remain within a $\pm 5\%$ band around the final $-20~\textrm{A}$ level, and undershoot was taken as the maximum excursion below that final current level during the transient. Experimentally, the battery current changed from $+20~\textrm{A}$ to $-20~\textrm{A}$ with a measured settling time of approximately $14.0~\textrm{ms}$. A peak current undershoot of $3.0~\textrm{A}$ below the final $-20~\textrm{A}$ level was observed, corresponding to approximately $15\%$, while the DC-link voltage exhibited a maximum transient deviation of $2.0~\textrm{V}$ and settled back to $30.0~\textrm{V}$. For the corresponding simulated reversal, the battery-current settling time was $12.0~\textrm{ms}$, the peak undershoot was $2.5~\textrm{A}$ below the final current level (approximately $12\%$), and the maximum DC-link deviation was $1.5~\textrm{V}$. The measured and simulated transients therefore show the same reversal direction, bounded DC-link excursion, and qualitatively similar damping behavior, which supports local trend-level transient agreement under the reported laboratory conditions. The quantified comparison is summarized in Table 12. This comparison should be interpreted as local, trend-level agreement with the corresponding simulation rather than as pointwise waveform matching.

The experimental observations therefore indicate laboratory-scale feasibility of the cascaded topology and the hierarchical controller under the reported prototype conditions. The reported experiments span one nominal G2V operating point, one nominal V2G operating point, and one commanded G2V$\rightarrow$V2G reversal case. Within this tested set, the measured DC-link and battery-side variables remained bounded and the intended power-flow direction was achieved in both steady-state modes and during the reported transition. At the same time, the present results should be interpreted as hardware validation under the reported operating conditions rather than as complete characterization across all power levels, battery chemistries, loading conditions, or operating envelopes.

Figure 14 makes the battery-side response explicit during the reported operating transition. In the charging interval, $I_o$ remains positive while $V_o$ remains close to the nominal battery-side level. During the commanded reversal, the current changes sign and settles to the reported discharge level, whereas the battery-side voltage remains bounded throughout the transition. The figure therefore complements Figs. 12 and 13 by directly showing the measured battery-side variables during charging and discharging.

**Table 12:** Comparison of the reported EXP-4 G2V$\rightarrow$V2G transient between experiment and simulation.

Case	$t_s$ (ms)	Undershoot	$\Delta V_s$ (V)	$V_s$ final (V)	Oscillation
Experiment (EXP-4)	14.0	$3.0~\textrm{A}$ ($\approx 15\%$)	2.0	30.0	None sustained
Simulation	12.0	$2.5~\textrm{A}$ ($\approx 12\%$)	1.5	30.0	None sustained

To complement the waveform plots, the three-run repeatability results for the nominal G2V operating point are summarized in Table 13, the steady-state power-balance estimates are reported in Table 14, and the steady-state comparison between analytical targets, simulation, and experiment is given in Table 15. Under the nominal G2V operating point, the measured values were $V_s=29.5~\textrm{V}$, $V_o=23.8~\textrm{V}$, $I_{Lk,pk}=8.5~\textrm{A}$, $I_{in,\textrm{avg}}=8.6~\textrm{A}$, and $I_o=20.0~\textrm{A}$. In V2G operation, the measured battery-side current reversed to approximately $-15.2~\textrm{A}$ while the DC-link voltage remained close to $30~\textrm{V}$. Across three repeated nominal G2V runs, the standard deviations remained small, indicating good operating-point repeatability under the reported laboratory conditions. The combined uncertainty values in Table 13 were computed by root-sum-square combination of the run-to-run standard deviation and the stated instrument uncertainty, i.e.,

$$\begin{aligned} u_c=\sqrt{u_{\textrm{instr}}^2+\textrm{SD}^2}. \end{aligned}$$

The input power was estimated as $P_{in}=V_{in,\textrm{avg}}I_{in,\textrm{avg}}$. For G2V operation, the battery-side power was estimated as $P_o=V_o I_o$. For V2G operation, the reported battery-side power magnitude is $|V_o I_o|$, and the sign of $I_o$ indicates reverse power flow from the battery toward the source. These values are intended as first-order electrical consistency checks based on averaged measured quantities rather than as a calorimetric efficiency characterization.

**Table 13:** Repeatability and uncertainty analysis for the nominal G2V operating point (three runs).

Quantity	Run 1	Run 2	Run 3	Mean	SD	Instr. unc.	Comb. unc.	Comment
$V_s$ (V)	29.4	29.6	29.5	29.5	0.1	$\pm 0.3$	$\pm 0.32$	Stable DC link
$V_o$ (V)	23.7	23.9	23.8	23.8	0.1	$\pm 0.2$	$\pm 0.22$	Battery-side ripple small
$I_{Lk,\textrm{pk}}$ (A)	8.4	8.6	8.5	8.5	0.1	$\pm 0.3$	$\pm 0.32$	Consistent leakage current
$I_{in,\textrm{avg}}$ (A)	8.5	8.6	8.7	8.6	0.1	$\pm 0.2$	$\pm 0.22$	Matches corrected power balance
$I_o$ (A)	19.8	20.1	20.0	20.0	0.15	$\pm 0.2$	$\pm 0.25$	Smooth charging current

**Table 14:** Steady-state power-balance and estimated efficiency check for the reported closed-loop operating cases. For G2V, $P_o=V_o I_o$ and the reported efficiency is the ratio of battery-side power to source-side power. For V2G, the reported battery-side quantity is the magnitude $|V_o I_o|$, while the negative sign of $I_o$ indicates reverse power flow from the battery toward the source; the reported efficiency is computed as source-side received power divided by battery-side delivered power magnitude. These values are intended as first-order electrical consistency checks based on averaged measured quantities rather than as calorimetric efficiency measurements.

Case	Mode	$\mathbf {V_{in}}$ (V)	$\mathbf {I_{in}}$ (A)	$\mathbf {P_{in}}$ (W)	$\mathbf {V_o}$ (V)	$\mathbf {I_o}$ (A)	$\mathbf {P_o}$ (W)	Eff. (%)
CL-SIM-1	G2V	60	8.5	510	24.1	20.8	501.0	98.2
EXP-2	G2V	60	8.6	516	23.8	20.0	476.0	92.2
EXP-3	V2G	60	6.0	360	24.0	−15.2	364.8	98.7

**Table 15:** Steady-state nominal-point comparison of analytical target, simulation, and experiment for the reported G2V operating condition. This table is intended as a local operating-point comparison rather than as general validation of the converter model over all operating conditions.

Quantity	Analyt.	Sim.	Exp.	$\mathbf {\Delta _s}$	%	$\mathbf {\Delta _e}$	%	Exp. SD
$V_s$ (V)	30.0	30.0	29.5	0.0	0.0	−0.5	−1.7	0.1
$V_o$ (V)	24.0	24.1	23.8	+0.1	+0.4	−0.2	−0.8	0.1
$I_{Lk,\textrm{pk}}$ (A)	9.0	9.2	8.5	+0.2	+2.2	−0.5	−5.6	0.1
$I_{in}$ (A)	8.4	8.5	8.6	+0.1	+1.2	+0.2	+2.4	0.1
$I_o$ (A)	20.0	20.8	20.0	+0.8	+4.0	0.0	0.0	0.15

**Table 16:** Summary of the reported experimental operating points of the laboratory prototype.

Case	Mode	$V_s$ (V)	$V_o$ (V)	$I_o$ (A)	$t_s$ (ms)	$\Delta V_s$ (V)	Interpretation
EXP-2	Nominal G2V steady state	29.5	23.8	20.0	–	–	Forward-power operating point used for steady-state validation.
EXP-3	Nominal V2G steady state	30.2	24.0	−15.2	–	–	Reverse-power operating point confirming bidirectional operation.
EXP-4	G2V$\rightarrow$V2G reversal	30.0 final	–	$+20\rightarrow -20$	14.0	2.0	Measured mode-reversal transient with bounded DC-link excursion.

**Table 17:** Qualitative comparison of the present laboratory prototype with representative bidirectional converter studies already cited in this manuscript.

References	Topology/application	Isolated	Bidirectional	Control emphasis	Main distinction relative to this work
^9^	Standalone DAB battery charging	Yes	Yes	SPS closed-loop charging control	Charging is performed within the DAB stage itself; no downstream Čuk stage and no explicitly coupled six-state cascaded model are considered.
^10^	DAB-based DC fast-charging interface	Yes	Yes	Charger-level control and implementation	Emphasis is placed on charger realization rather than on unified cascaded DAB–Čuk control-oriented modeling.
^11^	DAB-based EV charger	Yes	Yes	Modulation and loss-reduction strategy	Focus is on single-stage DAB charger operation rather than downstream current conditioning through a cascaded stage.
^12^	Multifunctional onboard charger	Yes	Yes	Multifunctional charger control	Addresses charger architecture and functionality rather than explicit intermediate-DC-link coupling in a cascaded DAB–Čuk interface.
^17^	Integrated three-port converter	Mixed	Application dependent	Topology and control synthesis	Represents a broader multiport energy interface rather than a dedicated isolated cascaded DAB–Čuk battery charger.
This work	Cascaded DAB–Čuk bidirectional battery charger	Yes	Yes	Hierarchical SPS + duty-ratio PI	Combines unified averaged/small-signal modeling, hierarchical regulation, and local bidirectional hardware validation for a cascaded isolated interface.

## Conclusion

This paper presented a control-oriented modeling and validation study of a cascaded DAB–Čuk converter for bidirectional battery-charging applications. A unified large-signal averaged model and a corresponding six-state small-signal representation were developed to describe the coupled dynamics introduced by the DAB-regulated intermediate DC link and the downstream converter stage. On this basis, a hierarchical dual-loop control structure was implemented, in which SPS modulation governed the DAB stage and duty-ratio control governed the downstream Čuk converter.

The reported simulation and laboratory-scale experimental results demonstrated controlled bidirectional operation under the tested G2V and V2G conditions. At the nominal G2V operating point, the analytical/simulation/experimental values of the intermediate DC-link voltage were 30.0/30.0/29.5 V, the battery-side voltage was 24.0/24.1/23.8 V, and the leakage-current peak was 9.0/9.2/8.5 A. In addition, the reported bidirectional reversal case showed bounded DC-link excursion and successful current-polarity reversal without sustained oscillation. Taken together, these results support the usefulness of the proposed unified modeling framework as a local control-oriented description of the cascaded converter under the reported laboratory operating conditions.

The present evidence should nevertheless be interpreted as operating-point-specific laboratory validation rather than as full operating-range dynamic verification of the converter model or broad charger qualification. Future work should therefore address broader operating envelopes, parasitic-inclusive modeling, extended transient model-to-hardware comparison, and higher-power validation.

## Data Availability

Data supporting the findings of this study are available from the corresponding author upon reasonable request. The shared material includes the simulation parameter set, controller gains, representative processed experimental measurements, and representative oscilloscope traces used for the steady-state and transient results reported in the manuscript.

## Appendix A Downstream Čuk switched-model averaging

For completeness, the downstream Čuk stage is modeled under CCM using the ON and OFF switched submodels given in Eqs. (14)–(17) and (18)–(21), respectively. Under the ideal-switching and CCM assumptions adopted in this manuscript, the averaged model is obtained by weighting the ON and OFF state equations over one PWM period according to the duty ratio $D_{S1}$. This standard state-space averaging step yields the duty-affine representation of the downstream Čuk dynamics and leads directly to the explicit averaged relations reported in Eqs. (24)–(27). Averaging these two submodels over one PWM period with duty ratio $D_{S1}$ gives

$$\begin{aligned} \dot{{\mathbf{x}}}_{\textrm{Cuk}} = D_{S1}\,{\mathbf{f}}_{\textrm{on}}({\mathbf{x}}_{\textrm{Cuk}},v_s) + (1-D_{S1})\,{\mathbf{f}}_{\textrm{off}}({\mathbf{x}}_{\textrm{Cuk}},v_s), \end{aligned}$$

where ${\mathbf{f}}_{\textrm{on}}(\cdot )$ and ${\mathbf{f}}_{\textrm{off}}(\cdot )$ denote the vector fields defined by the corresponding ON and OFF switched equations. The resulting averaged model preserves the control dependence on $D_{S1}$ while removing switching-period detail, which is appropriate for the local control-oriented analysis pursued in the main text. Linearization of those averaged equations about $(\bar{{\mathbf{x}}},{\bar{D}}_{S1})$ gives the Jacobian-based perturbation form used in Eq. (36).

## Code availability

The MATLAB/Simulink models and embedded-control implementation details used in this study are available from the corresponding author upon reasonable request. The shared material includes the Simulink model files, model parameter settings, and controller implementation settings sufficient to reproduce the reported simulation cases.

## Declarations

### Use of AI tools

During the preparation of this manuscript, the authors used generative AI tools only for language editing, grammar correction, and readability improvement. All technical content, verification, and final manuscript preparation were reviewed and approved by the authors.
